# Family traditions, accessibility and cost shape the continued reliance on *Cajanus cajan* and *Azadirachta indica* for malaria and typhoid despite prescription medicines in urban and rural Nsukka, Nigeria

**DOI:** 10.1186/s12906-026-05426-y

**Published:** 2026-06-04

**Authors:** Emmanuel E. Osayi, Dilibe C. Urama, Angela N. Amujiri, Ruth A. Agyo, Abdifatah Ahmed A. Afyare, Felix A. Andong

**Affiliations:** 1 Department of Plant Science and Biotechnology, Faculty of Biological Sciences, University of Nigeria, Nsukka, Enugu State Nigeria; 2 Department of Zoology and Environmental Biology, Faculty of Biological Sciences, University of Nigeria, Nsukka, Enugu State Nigeria; 3 AP Leventis Ornithological Research Institute, Faculty of Natural Sciences, University of Jos, Jos, Plateau State Nigeria; 4 Department of Public Veterinary, Public Health and Preventive Medicine, College of Veterinary Medicine, Joseph Sarwuan Tarka University, Makurdi, Nigeria; 5 Department of Social Sciences, Faculty of Economics and Management Science, Salaam University, Mogadishu, Somalia

**Keywords:** *Cajanus cajan*, *Azadirachta indica*, Malaria and typhoid management, Herbal medicine use, Socio-cultural determinants, Urban and rural healthcare practices

## Abstract

Despite the availability of conventional drugs, the use of medicinal plants for typhoid and malaria remains widespread in Nigeria. This study investigated the indigenous use of *Cajanus cajan* (pigeon pea) and *Azadirachta indica* (neem) in rural and urban Nsukka. We assessed: (i) socio-demographic factors influencing knowledge and use; (ii) the association between treatment practices and perceived efficacy; and (iii) socio-economic drivers of continued reliance on these herbal remedies. A cross-sectional survey of 400 respondents selected via stratified random sampling (200 urban, 200 rural) was conducted using structured questionnaires, where the primary outcome of this study was current herbal use, while knowledge was treated as a secondary outcome. Data were analyzed using generalized linear models (GLMs) and multinomial logistic regression (MLR) in *R* Statistical Software (version 4.3.0). Results indicated that rural residents relied more on herbal remedies due to accessibility, affordability, and family traditions, while urban respondents, despite higher education levels, also used herbal extracts and often alternated them with prescription drugs. Residence, occupation, and age were significant predictors of knowledge and use (*P* < 0.05), whereas education was not. Nonetheless, perceived efficacy and high prescription costs further sustained herbal use. While these findings highlight socio-cultural and economic factors sustaining herbal medicine use, they also support the need for public health education and integration of evidence-based traditional practices into primary healthcare to ensure safer use. However, the cross-sectional design limits causal inference. Future studies should combine questionnaire-based surveys with laboratory-based assessments.

**Supplementary Information:**

The online version contains supplementary material available at 10.1186/s12906-026-05426-y.

## Introduction

Limited access to affordable healthcare, high personal healthcare costs and uneven distribution of medical facilities remain persistent challenges in many low and middle income countries [1]. Within this context, healthcare-seeking behavior is best understood through the framework of health pluralism, where individuals simultaneously engage biomedical, traditional and self-care systems. Closely related is medical syncretism, which reflects the blending of different therapeutic traditions in response to social, cultural and economic realities [2, 3]. These frameworks emphasize that the continued use of herbal medicine is not a deviation from modern healthcare, but a rational response to structural barriers created by cost, accessibility and perceived quality of care [4].

Herbal medicine, as a component of complementary and alternative medicine, remains widely used across both developing and high income countries [5]. In Nigeria, medicinal plants and traditional healing practices play a significant role in primary healthcare delivery, supported by local knowledge systems and the expertise of herbal medicine practitioners [6–10]. These practices provide accessible, affordable and culturally accepted therapeutic options, while also contributing to pharmaceutical research through the identification of bioactive compounds [11].

Among commonly utilized species, *Cajanus cajan* (Fabaceae) is frequently used in the management of typhoid related symptoms [12], whereas *Azadirachta indica* (Meliaceae) is widely employed in malaria treatment due to its documented antiplasmodial activity, which inhibits microgametogenesis and disrupt the parasite development [13, 14]. The sustained use of these plant based therapies reflects their local availability and affordability, as well as strong cultural beliefs, perceived efficacy, and intergenerational knowledge transfer.

Despite their widespread use, empirical evidence on patterns of herbal medicine utilization remains limited, particularly regarding dosage practices, perceived effectiveness and determinants of use. This gap is especially relevant for malaria and typhoid, which are commonly managed at the community level through self-diagnosis and self-medication. Since most reports of malaria typhoid co-infection are sometimes symptom based rather than laboratory confirmed, this practice leads to significant symptom overlap and often results in overestimation of true co-infection prevalence [15]. Nonetheless, focusing on the self-reported co-occurrence of malaria and typhoid like symptoms remains vital because it provides insight into the community’s perceived need and the lived experience of illness that influences the use of multiple healthcare options that include the combination of herbal and biomedical treatments.

Nonetheless, such health seeking practices are shaped by socio cultural and environmental factors such as family traditions, community norms and informal knowledge networks, which influence perceptions of treatment efficacy and safety [16]. Also, structural determinants such as income, education and proximity to healthcare facilities or natural plant sources influence access to and preference for specific treatment options. Even in urban settings where biomedical services are more available, individuals continue to engage in pluralistic healthcare practices as they combine or alternate between different treatment systems depending on perceived need and accessibility.

However, despite the growing body of literature on herbal medicine use, there remains a limited understanding of how these socio cultural, structural and perceptual factors interact specifically in the context of malaria and typhoid like illnesses in Nsukka, Nigeria. In particular, few studies have simultaneously examined knowledge, treatment choices and perceived efficacy of *Cajanus cajan* and *Azadirachta indica* within a pluralistic healthcare framework, especially when considering differences between rural and urban populations. This gap limits evidence based understanding of why these medicinal plants remain widely used despite the availability of biomedical alternatives.

Against this backdrop, this study investigates the factors influencing the indigenous use of Cajanus cajan and *Azadirachta indica* for the management of malaria and typhoid like illnesses in rural and urban areas of Nsukka, Nigeria. Specifically, the study aims to: (i) assess the demographic and socio cultural determinants of knowledge and use of these medicinal plants; (ii) examine the association between treatment modality (herbal, biomedical, or combined) and perceived efficacy; and (iii) evaluate the influence of socio economic and accessibility factors on treatment choices. These perspectives are complemented by the health belief model, which explains how individual perceptions of illness, treatment effectiveness and access influence health seeking decisions.

More so, our study situates herbal medicine use within the frameworks of health pluralism, medical syncretism and structural healthcare constraints, which provides a theoretically grounded understanding of treatment practices and generates evidence relevant to healthcare integration and policy development in Nigeria. Furthermore, this study is grounded in the Health Belief Model [17, 18] and medical pluralism [2, 3].

As such, previous evidence suggests that rural residence, lower educational attainment, high healthcare costs, and limited access to formal medical facilities may increase reliance on herbal medicine [4, 19]. In addition, family and intergenerational traditions play a central role in shaping trust in indigenous knowledge systems and influencing dosage practices and perceived efficacy [16]. Within pluralistic healthcare systems, treatment choices are often influenced by perceived effectiveness, cultural acceptability and accessibility [5], and also, proximity to natural plant resources and healthcare facilities may influence treatment preferences by altering perceived cost and ease of access [4].

Based on this, the study tests the following hypotheses: that (i) socio-demographic factors (education, residence, age, and occupation) are significantly associated with knowledge and use of *C. cajan* and *A. indica*; (ii) family and intergenerational traditions are significantly associated with dosage practices and perceived efficacy of herbal medicine [16]; (iii) treatment modality (herbal, biomedical, or combined) is significantly associated with perceived effectiveness [5]; and (iv) accessibility factors, including proximity to plant sources and cost of prescription medicines, are significantly associated with reliance on herbal medicine [4].

## Materials and methods

### Study area

**Fig. 1:**
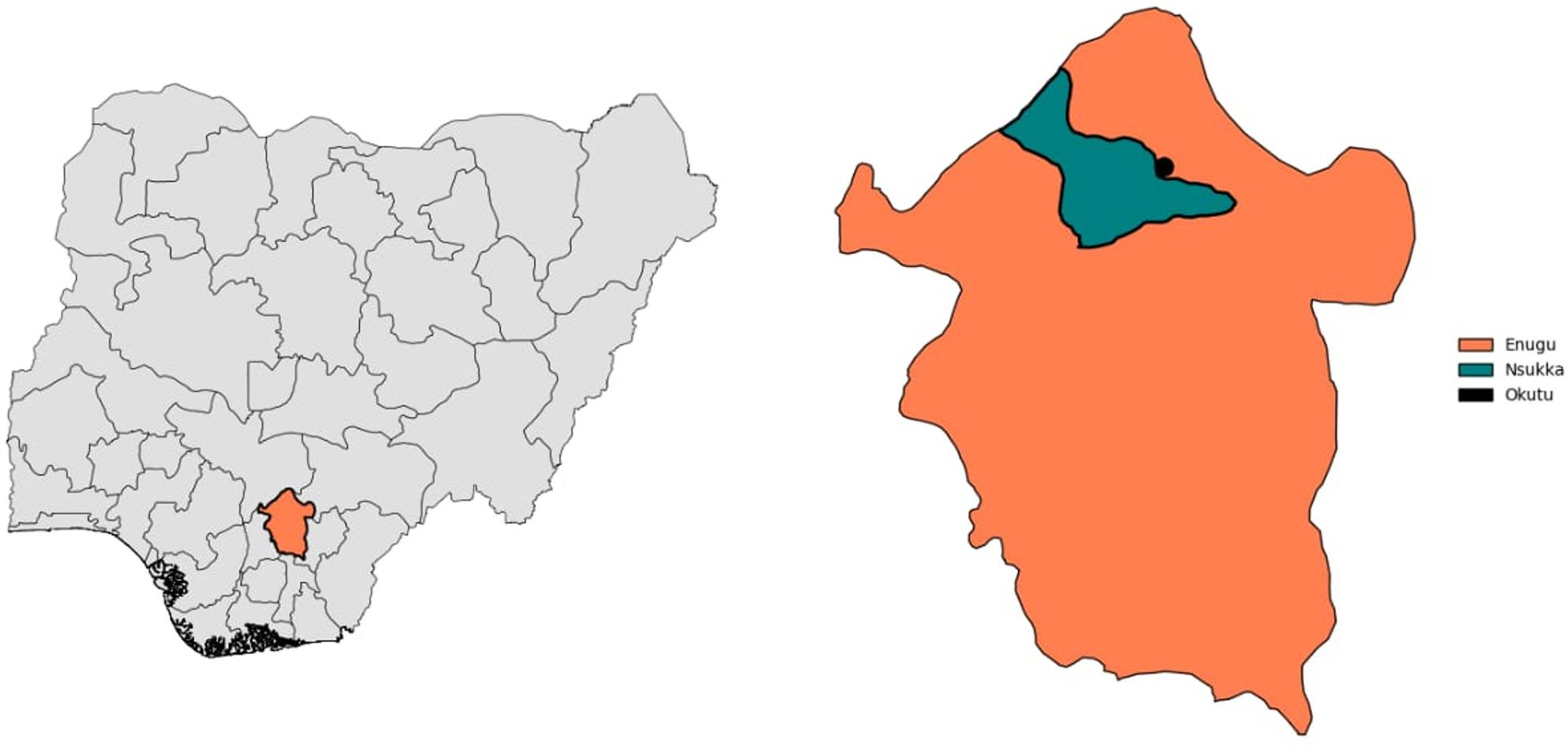
Map of Nigeria highlighting Enugu State (left) and the study area (right), showing the sampling locations in Nsukka (urban) and Okutu (rural) communities

The study was conducted in 2025 across urban and rural communities within the Nsukka Local Government Area (LGA), Enugu State, Nigeria (6°50′N, 7°22′E) (Fig. 1). Nsukka is a predominantly Igbo speaking region covering approximately 5,545 km², with a population estimated at 309,633 [20, 21]. The demographic structure is uniquely influenced by the University of Nigeria, Nsukka, which drives significant temporary migration of students and visitors [22]. The region reflects a mixed socio-economic population structure typical of southeastern Nigeria, where literacy levels and educational attainment are generally higher in urban and semi urban settlements compared to rural communities, which are largely characterized by lower literacy levels and greater reliance on subsistence agriculture and informal trading. Employment patterns similarly vary across the study area, with urban residents predominantly engaged in civil service, education, and small scale business activities, while rural inhabitants are mainly farmers and informal herbal practitioners [23]. These socio-economic characteristics help explain observed differences in healthcare access and the continued reliance on both traditional and biomedical treatment systems within the study population. This academic presence creates a distinct socio economic contrast, where urban and semi urban settlements exhibit higher literacy and educational attainment, while surrounding rural communities remain largely dependent on agriculture and small scale trading, often with limited access to formal healthcare infrastructure [24]. Consequently, the area represents a complex rural urban interface in which civil service employment and traditional farming coexist [23], providing an important context for the continued integration of traditional medicinal plants alongside biomedical healthcare systems.

In addition to its socio demographic complexity, the topography and vegetation of Nsukka further influence the availability and accessibility of medicinal plant resources. The region is bordered by Kogi and Benue States to the north and Anambra State to the west [25], and experiences a tropical climate characterized by a distinct wet season (March to October) and a short dry season (November to February) [26]. Rainfall typically peaks in September, with mean annual precipitation of approximately 1,500 mm and temperatures ranging between 22 °C and 30 °C. These climatic conditions support a transitional vegetation zone between woodland tall grass savannah and lowland rainforest, resulting in a rich diversity of plant species traditionally used for medicinal purposes.

As such, several plant species such as *Mangifera indica* (mango) and *Carica papaya* (pawpaw), are commonly utilized locally for therapeutic purposes [27, 28]. More so, findings from the pre survey identified *Azadirachta indica* (neem) and *Cajanus cajan* (pigeon pea) as the most frequently used medicinal extracts for managing malaria and typhoid in the study area. Previous ethnobotanical studies in the region further confirm that various plant parts, particularly leaves, bark and roots, are also commonly prepared as decoctions and infusions, thereby reflecting longstanding indigenous knowledge systems and traditional healthcare practices [29].

### Selection of urban and rural areas

The socio-economic differences between the urban and rural communities of Nsukka, especially in terms of education, income and access to healthcare usually affects the continued reliance on herbal medicine as either a primary or complementary healthcare option [9]. Nsukka Urban, which comprises the Local Government Area (LGA) headquarters and the University of Nigeria, serves as the major commercial hub of the region and was therefore designated as the urban study area [30]. During the pre-survey, it was observed that a substantial number of people in the Nsukka Urban community make use of herbal remedies for typhoid and malaria.

Conversely, in the pre-survey conducted to identify rural areas that still depend on herbal medicines for treating common ailments such as malaria and typhoid, Okutu-Nsukka, which is a community bordering Kogi State and characterized by extensive forested areas used for the extraction of wild herbs, was found to remain highly dependent on plant-based treatments. This continued reliance is largely attributed to the considerable distance from conventional healthcare facilities, which are primarily concentrated in the Nsukka Urban area [21].

### Study design and questionnaire administration

A cross-sectional survey design was employed to investigate indigenous knowledge, usage patterns and socio-cultural determinants of *Cajanus cajan* and *Azadirachta indica* utilization. Structured questionnaires were used for data collection, where current herbal use was operationally defined as the primary outcome, while indigenous knowledge was treated as a secondary outcome. Nonetheless, before the main survey, a one-week pre-survey was conducted to pilot-test the questionnaire and refine items regarding plant knowledge, preparation methods, dosage and perceived efficacy. Our sampling frame consisted of residential household lists derived from the National Population Commission census data [20], updated by local community leaders in Nsukka Urban and Okutu-Nsukka.

Basically, our study utilized a stratified random sampling method to ensure representative data from both demographic settings. The study area was stratified into two primary strata: urban (Nsukka Urban) and rural (Okutu-Nsukka). Within these strata, we employed a systematic random walk recruitment process; starting from a central landmark in each stratum, every 5th household was approached by trained research assistants (final-year undergraduate students) to invite participation. To ensure data relevance, specific inclusion criteria required participants to be adults (≥ 18 years), residents for at least six months, and possess prior knowledge or personal experience with the medicinal use of *C. cajan* or *A. indica*. Individuals below the age of 18, temporary visitors, or those unwilling to provide informed consent were excluded.

Afterwards, our sample size was determined using Slovin’s formula: $\mathrm{n}=\mathrm{N}/[1+\mathrm{N}\left(\mathrm{e}\right)^2]$, where n was the required sample size, N was the total population of eligible respondents, and e is the margin of error (5%). Based on the National Population Commission [20] records for the study area (*N* ≈ 309,633), a minimum of 400 respondents was required to ensure statistical representativeness. Nonetheless, to account for potential data loss, incomplete responses, or non-participation, 500 questionnaires were produced and equally distributed (250 per stratum). This equal distribution was selected over proportional allocation to minimize the standard error of the difference between strata means [31].

### Data collection

The survey was interviewer-administered to ensure high data quality, minimize missing values and accommodate varying literacy levels. Questionnaires were administered face-to-face in either English or the local Igbo dialect, depending on respondents’ preferences. Data were collected weekly between 10:00 a.m. and 2:00 p.m., when respondents were most accessible. In the field, research assistants approached 435 households, achieving a 91.9% household-level consent and completion rate (400/435). In some households, however, multiple eligible respondents participated, resulting in 441 completed questionnaires. Reasons for non-participation primarily included lack of time or interest in the study. The structured questionnaire captured comprehensive data across five key domains: (i) demographic information, including age, gender, marital status, parity, education, and occupation; (ii) knowledge and usage patterns regarding *C. cajan* and *A. indica*, specifically awareness, frequency, purpose (prevention vs. treatment), and perceived efficacy; (iii) socio-cultural factors, such as sources of indigenous knowledge, family history of plant use, and cultural beliefs; (iv) safety awareness, covering experiences with side effects and health risk perceptions; and (v) co-occurrence practices, focusing on dosage patterns when managing typhoid and malaria symptoms simultaneously.

Following the data collection phase, while 435 households were successfully recruited, the inclusion of multiple eligible respondents within certain households resulted in a raw retrieval of 441 valid questionnaires (239 from the urban stratum and 202 from the rural stratum). To maintain a balanced design for comparative statistical analysis and prevent demographic bias, the final sample was standardized to 200 respondents per stratum (*N* = 400 total). This balancing ensured that both groups had equal weight during the application of generalized linear models (GLMs) and multinomial logistic regression, thereby improving the reliability of the comparative results. Post-hoc power analysis confirmed that this balanced sample provides a power greater than 0.80 to detect a medium effect size at an alpha level of 0.05.

### Questionnaire validation

The structured questionnaire was subjected to face and content validation by a panel of three experts including ethnobiologists and ecologists from the Departments of Plant Science and Biotechnology and Zoology and Environmental Biology at the University of Nigeria, Nsukka. This review ensured that the items adequately addressed the research objectives. Following this, a pilot study was conducted among 20 respondents in a non-sampled community to test for item clarity and cultural sensitivity.

The reliability of the instrument was statistically verified using Cronbach’s alpha: α = (k / (k − 1)) * (1 - (Σ σ²_i_ / σ²ₜ)), where α is the Cronbach’s alpha (internal consistency), k is the number of items in the scale, Σ σ²_i_ is the sum of the variances of each individual item, and σ²ₜ is the total variance of the sum of the scores. This calculation yielded a coefficient of 0.82, surpassing the recommended threshold of 0.70 for robust social science research.

Nonetheless, to accommodate varying literacy levels and ensure data accuracy, the survey was interviewer-administered. The questionnaire was available in both English and the local Igbo dialect. To maintain linguistic and conceptual consistency, a back-translation protocol was employed; the original English version was translated into Igbo by a language specialist and subsequently back-translated into English by an independent researcher. Any discrepancies in the translation of plant-related terminology or disease symptoms were reconciled prior to the final administration.

### Statistical design and data analysis

All data were analyzed in *R* Statistical Software (version 4.3.0) [32]. To ensure the accuracy of our estimates, all regression results are reported as Odds Ratios (OR) accompanied by 95% Confidence Intervals (CI). To minimize the risk of Type I errors associated with multiple comparisons, a Bonferroni correction was applied to all p-value thresholds. More so for data integrity, the interviewer-administered format ensured high completion rates; however, in the rare instance of incomplete records (< 2%), missing data were handled using listwise deletion to ensure only complete cases were included in the final models.

A descriptive statistic was used to summarize participants’ responses. To examine the demographic and socio-cultural factors influencing knowledge and use of *C. cajan* and *A. indica* for treating malaria, typhoid, and their co-infections, a generalized linear model (GLM) with a binomial error structure and logit link was fitted. The model included education (none, primary, secondary, tertiary), gender (male, female), residence (rural, urban), age group (18–35, 36–53, 54–72, > 72), occupation (civil servant, business, farmer, herbalist), and the interaction between age and education as fixed effects, while knowledge of herbal use (yes/no) was the response variable. The interaction term between age group and education level was included in the model to account for the possibility that the effect of education on knowledge and use of the herbs may vary across different age groups. We assumed that younger participants with higher education may have different treatment practices compared with older participants with the same education level.

To examine our second objective, which is the relationship between treatment practices (herbal, prescription, or both) and the perceived efficacy or frequency of use of *C. cajan* and *A. indica*, we fitted a multinomial logistic regression model using the nnet package. Our response variable was treatment practice, and predictor variables included perceived efficacy, frequency of use, age group, gender, residence, occupation, and education. Also, we included the interaction between age and perceived efficacy. This interaction was to account for the possibility that the effect of perceived efficacy on treatment choice may vary across different age groups. We assumed that younger participants who perceive the herbs as highly effective may choose different treatment practices compared with older participants with the same perception of efficacy.

To examine our last objectives, which is assessing the socio-economic and accessibility factors influencing the continued reliance on herbal remedies, a GLM with a binomial error structure and logit link was fitted. The model included gender (male, female), age group (18–35, 36–53, 54–72, > 72), education level (none, primary, secondary, tertiary), residence (rural, urban), proximity to source (far/near), perceived accessibility of herbal remedies (yes/no), cost of prescription medicines (yes/no), poverty status (yes/no), cultural beliefs (yes/no), family influence (yes/no), perceived efficacy (low, moderate, high), treatment sought (herbal, prescription/both), and the interaction between proximity and accessibility as fixed effects. The response variable was whether respondents reported using herbal remedies (yes/no). The interaction term between proximity and accessibility was included to account for the possibility that the effect of accessibility on herbal remedy use may differ depending on how close respondents reside to herbal sources. Assuming that, respondents living closer to herbal resources may rely more on herbs if they are easily accessible compared with those living farther away.

Model selection involved a backward removal of non-significant terms (adjusted *p* < 0.05) starting with the interaction. To explore the importance of specific variables in explaining our outcome, irrespective of statistical significance, and to evaluate the practical relevance of particular predictors for herbal remedy use in our study population, we used partial η² to assess effect sizes based on conventional benchmarks. These benchmarks classify effect sizes as ~ 0.01 (small or weak), ~ 0.06 (medium) and ~ 0.14 (large or strong) [31, 33]. Partial η² values are reported with their corresponding 95% Confidence Intervals to describe the precision of the effect size estimates.

## Results

### Socio-demographic characteristics of respondents

Our study showed that among the 400 respondents (200 urban; 200 rural), urban areas had more females (55%), whereas rural areas were predominantly male (70%). The 36 to 53 age group was more common in rural areas (50%), while older adults aged 54 to 71 years were more represented in urban areas (43%). Marriage rates were higher among rural respondents than urban respondents (81% vs. 54%). Urban participants were mainly civil servants (40%) and business owners (33%), whereas rural respondents were largely farmers (44%) and herbalists (19%). Educational attainment also differed markedly: 65% of urban respondents had tertiary education compared to 19% in rural areas, where primary (40%) and secondary (27%) education levels were most common (Table 1).

**Table 1:** Respondents’ demographic information

Variables	Urban (*n* = 200)	Rural (*n* = 200)
Gender		
Male	89 (44.5%)	139 (69.5%)
Female	111 (55.5%)	61 (30.5%)
Age Range		
18–35	19 (9.5%)	22 (11.0%)
36–53	73 (36.5%)	101 (50.5%)
54–71	87 (43.5%)	61 (30.5%)
72 and above	21 (10.5%)	16 (8.0%)
Marital Status		
Yes	109 (54.5%)	162 (81.0%)
No	91 (45.5%)	38 (19.0%)
Number of Children (if married)	**(*n*** = **109)**	**(*n*** = **162)**
None	0 (0.0%)	0 (0.0%)
1–5	87 (79.8%)	108 (66.7%)
6–10	22 (20.2%)	54 (33.3%)
Occupation		
Civil servant	81 (40.5%)	5 (2.5%)
Business	65 (32.5%)	68 (34.0%)
Farmer	54 (27.0%)	88 (44.0%)
Herbalist	0 (0.0%)	39 (19.5%)
Level of Education		
None	3 (1.5%)	27 (13.5%)
Primary	21 (10.5%)	80 (40.0%)
Secondary	45 (22.5%)	55 (27.5%)
Tertiary	131 (65.5%)	38 (19.0%)

### Knowledge and experience of typhoid and malaria

We found that respondents’ experiences with typhoid and malaria differed by location. Rural residents reported higher malaria (41%) and typhoid (37%) than urban respondents (33% and 30%), while urban respondents reported higher co-infection (37%). Diagnosis practices indicated that both rural and urban areas relied more on symptoms (81% and 46%) than laboratory tests to confirm infections. Most urban respondents indicated fever (28%) and loss of appetite (27%) as primary symptoms, while rural respondents reported fever (34%) and headache (30%) (Table 2). Urban respondents reported higher use of prescription drugs (48%), while rural participants more frequently reported combined herbal and prescription use (47%). Herbal-only use was more commonly reported in urban areas (42%) than rural areas (30%), whereas rural respondents more frequently reported combined use of herbal and prescription treatments. Urban respondents reported using herbal remedies mainly for perceived efficiency (64%), whereas rural respondents reported affordability (51%) and accessibility (34%) as common reasons (Table 2). Prescription use in rural areas was associated with health practitioner recommendations (53%), while 47% of urban respondents reported affordability as a key factor. Among respondents using both treatment types, cost considerations were frequently reported in both rural and urban areas (63% and 45%) (Table 2).

**Table 2:** Indicating respondents’ knowledge about typhoid and malaria

Variables	Urban (*n* = 200)	Rural (*n* = 200)
Have you ever been diagnosed or believed to have:		
Typhoid	59 (30%)	75 (37%)
Malaria	67 (33%)	82 (41%)
Both	74 (37%)	43 (22%)
None	0 (0%)	0 (0%)
How do you confirm typhoid or malaria infection?		
Laboratory test	76 (38%)	31 (16%)
Symptoms only	92 (46%)	162 (81%)
Both	32 (16%)	7 (3%)
If based on symptoms, which do you usually experience?	**(*n*** = **92)**	**(*n*** = **162)**
Headache	19 (21%)	49 (30%)
Fever	26 (28%)	55 (34%)
Nausea	13 (14%)	0 (0%)
Loss of appetite	25 (27%)	33 (20%)
Body pains	9 (10%)	6 (4%)
Fatigue	0 (0%)	19 (12%)
Type of medication used for prevention or treatment:		
Herbal	84 (42%)	61 (30%)
Prescription	96 (48%)	45 (23%)
Both	20 (10%)	94 (47%)
If herbal, why do you prefer it?	**(*n*** = **84)**	**(*n*** = **61)**
Accessible	4 (4%)	21 (34%)
Affordable	3 (4%)	31 (51%)
Efficient	54 (64%)	2 (3%)
Compatible	21 (26%)	1 (2%)
Family recommendation	2 (2%)	6 (10%)
If prescription, why do you prefer it?	**(*n*** = **96)**	**(*n*** = **45)**
Accessible	12 (13%)	12 (27%)
Affordable	45 (47%)	4 (9%)
Efficient	22 (22%)	3 (7%)
Compatible	17 (18%)	2 (4%)
Recommended by health practitioner	0 (0%)	24 (53%)
If both, why do you use it more often?	**(*n*** = **20)**	**(*n*** = **94)**
Recommendation	7 (35%)	33 (35%)
Depends on severity	4 (20%)	2 (2%)
Cost-related choice	9 (45%)	59 (63%)

### Knowledge and use of *Cajanus cajan*

We observed that knowledge and use of *C. cajan* were higher in rural areas (86%) than urban areas (67%). Both rural and urban respondents reported using *C. cajan* for treating typhoid (91% and 53%). Knowledge of *C. cajan* use was reported to be associated with family history in rural areas (77%), while urban respondents more frequently reported recommendations (46%). Frequent use was reported in both urban and rural respondents (59% and 79%, respectively). Longer duration of use was more commonly reported in rural areas (61% reported 11–20 years) compared to urban respondents (50% reported 1–5 years). Rural respondents more frequently reported using the extract for treatment (49%), while urban respondents more commonly reported preventive use (90%). Combined use of *C. cajan* with prescription drugs was frequently reported in both settings (86% urban; 87% rural). Mixing with other herbs was also reported among urban (26%) and rural respondents (69%). Self-reported dosing patterns reported in both groups, with rural respondents more frequently reporting one cup twice daily (81%), while urban respondents more often reported half cup twice daily (45%). Reported side effects were higher among urban respondents compared to rural respondents (69% vs. 26%) (Table 3).

**Table 3:** Indicating respondents’ knowledge and use of *Cajanus cajan*

Variables	Urban	Rural
Do you have any knowledge about *Cajanus cajan*?	**(*n*** = **200)**	**(*n*** = **200)**
Yes	134 (67%)	172 (86%)
No	66 (33%)	28 (14%)
If yes, what is the plant mostly used for?	**(*n*** = **134)**	**(*n*** = **172)**
Malaria	29 (22%)	5 (3%)
Typhoid	71 (53%)	158 (91%)
Ulcer	9 (7%)	3 (1%)
Purgative	25 (18%)	6 (3%)
If used for typhoid, how did you acquire this knowledge?	**(*n*** = **71)**	**(*n*** = **158)**
Family	16 (23%)	122 (77%)
Recommendation	33 (46%)	3 (2%)
Books	3 (4%)	0 (0%)
School	9 (13%)	4 (3%)
Herbal practitioner	10 (14%)	29 (18%)
Frequency of *Cajanus cajan* use for typhoid:	**(*n*** = **71)**	**(*n*** = **158)**
Always	42 (59%)	125 (79%)
Rarely	13 (18%)	12 (8%)
Never	16 (23%)	21 (13%)
Duration of use (years):	**(*n*** = **42)**	**(*n*** = **125)**
1–5	21 (50%)	6 (5%)
6–10	16 (38%)	12 (9%)
11–20	2 (5%)	76 (61%)
Above 20	3 (7%)	31 (25%)
When is *Cajanus cajan* mainly used?	**(*n*** = **42)**	**(*n*** = **125)**
Prevention	38 (90%)	25 (20%)
Treatment	3 (7%)	61 (49%)
Both	1 (2%)	39 (31%)
How do you alternate between herbal and the prescribed drug?	**(*n*** = **42)**	**(*n*** = **125)**
*Cajanus cajan* for prevention, prescribed for treatment	36 (86%)	9 (7%)
*Cajanus cajan* for treatment, prescribed for prevention	4 (9%)	7 (6%)
Use both together	2 (5%)	109 (87%)
How is *Cajanus cajan* usually prepared?	**(*n*** = **42)**	**(*n*** = **125)**
Boiling leaves or roots (decoction)	21 (50%)	31 (25%)
Soaking (infusion)	10 (24%)	0 (0%)
Grinding with water	0 (0%)	6 (5%)
Mixing with other herbs	11 (26%)	86 (69%)
Other (specify)	0 (0%)	0 (0%)
Usual dosage or rate of consumption:	**(*n*** = **42)**	**(*n*** = **125)**
One cup twice daily	14 (33%)	101 (81%)
One cup once daily	9 (21%)	14 (11%)
Half cup twice daily	19 (45%)	10 (8%)
Duration per treatment cycle:	**(*n*** = **42)**	**(*n*** = **125)**
1–3 days	2 (5%)	42 (34%)
4–7 days	33 (78%)	18 (14%)
More than 7 days	7 (17%)	65 (52%)
Experienced side effects after using *Cajanus cajan*:	**(*n*** = **42)**	**(*n*** = **125)**
Yes	29 (69%)	33 (26%)
No	13 (31%)	92 (74%)

### Knowledge and use of *Azadirachta indica*

We found that knowledge and use of *A. indica* were higher in rural areas (74%) than urban areas (44%). Both settings reported malaria as the primary use (86% rural; 84% urban). Knowledge was reported to be associated with family sources in both rural (77%) and urban (66%) settings. Frequent use was reported in both groups (80% rural; 57% urban), with longer duration more commonly reported in rural respondents (57% reported 11–20 years) compared with urban respondents (52% reported 6–10 years). Both rural and urban respondents reported mainly using *A. indica* for treatment rather than prevention (69% and 55%). Combined use with prescription medicine was frequently reported in both groups (90% urban; 94% rural). Decoction was the most commonly reported preparation method (67% urban; 80% rural). Self-reported dosing patterns were more commonly reported as one cup twice daily in rural areas (91%), while urban respondents more frequently reported half cup twice daily (74%). Also, reported side effects were higher in rural respondents compared with urban respondents (54% vs. 38%) (Table 4).

**Table 4:** Indicating respondents’ knowledge and use of *Azadirachta indica*

Variables	Urban	Rural
Do you have any knowledge about *Azadirachta indica*?	**(*n*** = **200)**	**(*n*** = **200)**
Yes	88 (44%)	147 (74%)
No	112 (56%)	53 (26%)
If yes, what is the plant mostly used for?	**(*n*** = **88)**	**(*n*** = **147)**
Typhoid	3 (3%)	4 (3%)
Malaria	74 (84%)	127 (86%)
Both	11 (13%)	16 (11%)
If used for malaria, how did you acquire this knowledge?	**(*n*** = **74)**	**(*n*** = **127)**
Family	49 (66%)	98 (77%)
Recommendation	2 (3%)	27 (21%)
Books	0 (0%)	0 (0%)
School	9 (12%)	0 (0%)
Herbal practitioner	14 (19%)	2 (1%)
Frequency of *Azadirachta indica* use for malaria:	**(*n*** = **74)**	**(*n*** = **127)**
Always	42 (57%)	102 (80%)
Rarely	11 (15%)	25 (20%)
Never	21 (28%)	0 (0%)
Duration of use (years):	**(*n*** = **42)**	**(*n*** = **102)**
1–5	9 (21%)	14 (14%)
6–10	22 (52%)	19 (18%)
11–20	6 (14%)	59 (57%)
Above 20	5 (12%)	10 (10%)
When is *Azadirachta indica* mainly used?	**(*n*** = **42)**	**(*n*** = **102)**
Prevention	7 (17%)	33 (32%)
Treatment	29 (69%)	56 (55%)
Both	6 (14%)	13 (12%)
Have you alternated *Azadirachta indica* with prescribed medications?	**(*n*** = **42)**	**(*n*** = **102)**
Yes	38 (90%)	96 (94%)
No	4 (10%)	6 (6%)
If yes, how do you alternate them?	**(*n*** = **38)**	**(*n*** = **96)**
*Azadirachta indica* for prevention, prescribed for treatment	29 (76%)	12 (12%)
*Azadirachta indica* for treatment, prescribed for prevention	4 (10%)	51 (53%)
Use both together	5 (13%)	33 (34%)
How is *Azadirachta indica* usually prepared?	**(*n*** = **42)**	**(*n*** = **102)**
Boiling leaves or roots (decoction)	28 (67%)	82 (80%)
Soaking (infusion)	2 (5%)	0 (0%)
Grinding with water	1 (2%)	0 (0%)
Mixing with other herbs	11 (26%)	20 (20%)
Other (specify)	0 (0%)	0 (0%)
Usual dosage or rate of consumption:	**(*n*** = **42)**	**(*n*** = **102)**
One cup twice daily	31 (74%)	93 (91%)
One cup once daily	2 (5%)	6 (6%)
Half cup twice daily	9 (21%)	3 (3%)
Duration per treatment cycle:	**(*n*** = **42)**	**(*n*** = **102)**
1–3 days	16 (38%)	66 (65%)
4–7 days	19 (45%)	11 (10%)
More than 7 days	7 (17%)	25 (24%)
Experienced side effects after use:	**(*n*** = **42)**	**(*n*** = **102)**
Yes	16 (38%)	55 (54%)
No	26 (62%)	47 (46%)

### Co-occurrence of malaria and typhoid and treatment practices

Among respondents who reported using both herbal extracts, all participants reported a history of perceived malaria and typhoid-like symptoms, and it is important to note that these responses reflect self-reported experiences rather than laboratory-confirmed co-infection. During reported co-occurrence, both rural (67%) and urban (52%) respondents indicated combined use of *C. cajan* and *A. indica*, while others reported combining herbal remedies with hospital medicines (23% rural; 38% urban). Combined preparation methods were frequently reported in both settings (91% rural; 63% urban). Self-reported dosing patterns were commonly one cup once daily in rural areas (62%), while urban respondents more frequently reported half cup twice daily (86%).Reported symptom relief following use was high in both rural (99%) and urban (86%) respondents. Reported side effects during combined use were uncommon, with few urban cases (14%) and none reported in rural areas (Table 5).

**Table 5:** Respondents’ treatment practices during co-infection with typhoid and malaria

Variables	Urban	Rural
Have you ever experienced typhoid and malaria at the same time?	**(*n*** = **42)**	**(*n*** = **102)**
Yes	42 (100%)	102 (100%)
No	0 (0%)	0 (0%)
If yes, how do you usually treat both?	**(*n*** = **42)**	**(*n*** = **102)**
*Cajanus cajan* and *Azadirachta indica* together	22 (52%)	68 (67%)
*Cajanus cajan* only	2 (5%)	9 (9%)
*Azadirachta indica* only	2 (5%)	1 (1%)
Combine herbs with hospital drugs	16 (38%)	24 (23%)
Preparation method when combining herbs:	**(*n*** = **22)**	**(*n*** = **68)**
Separately	5 (22%)	4 (6%)
Together in same decoction	14 (63%)	62 (91%)
Sequentially (one after another)	3 (14%)	2 (3%)
Dosage when combining herbs:	**(*n*** = **22)**	**(*n*** = **68)**
One cup twice daily	2 (9%)	22 (32%)
One cup once daily	1 (5%)	42 (62%)
Half cup twice daily	19 (86%)	4 (6%)
Relief experienced when using both:	**(*n*** = **22)**	**(*n*** = **68)**
Yes	19 (86%)	67 (99%)
No	0 (0%)	0 (0%)
Partially	3 (14%)	1 (1%)
Who recommends the use of these herbs for co-occurrence?	**(*n*** = **22)**	**(*n*** = **68)**
Family	21 (95%)	68 (100%)
Herbalist	0 (0%)	0 (0%)
Friends	1 (5%)	0 (0%)
Self	0 (0%)	0 (0%)
Experienced side effects when using both herbs together:	**(*n*** = **22)**	**(*n*** = **68)**
Yes	3 (14%)	0 (0%)
No	19 (86%)	68 (100%)

### Factors influencing herbal medicine use

Herbal use in rural areas was reported to be associated with accessibility (38%) and cultural beliefs (28%), while urban respondents reported associations with family tradition (33%) and cultural beliefs (28%). A large proportion of rural respondents (85%) reported willingness to continue herbal use even if prescription drugs were cheaper, with responses associated with traditional belief (57%) and cost considerations (38%); similar patterns were reported among urban respondents (81%). Continued reliance on *C. cajan* and *A. indica* was reported alongside cultural practices (46% rural; 38% urban), with accessibility (39%) in rural areas and perceived safety (35%) in urban areas frequently mentioned. Proximity to plant sources was higher among rural respondents (81%) compared with urban respondents (7%). Awareness of potential side effects was high in both groups (100% urban; 95% rural) (Table 6). Respondents reported that increased awareness (62% urban) and drug subsidies (67% rural) may improve uptake of prescription medicines.

**Table 6:** Factors influencing respondents’ use of herbal remedies and suggested future awareness and safety measures

Variables	Urban (*n* = 42)	Rural (*n* = 102)
Factors influencing preference for herbal remedies:		
Cost	0 (0%)	2 (1%)
Accessibility	8 (19%)	52 (38%)
Cultural belief	12 (28%)	32 (28%)
Effectiveness	8 (19%)	15 (7%)
Family influence	14 (33%)	27 (26%)
Would you stop using herbal medicines if prescribed drugs were more affordable?		
Yes	31 (74%)	16 (15%)
No	11 (26%)	86 (85%)
If no, why do you still prefer herbal remedies?	**(*n*** = **11)**	**(*n*** = **86)**
Affordability	0 (0%)	2 (2%)
Cost-effective	0 (0%)	33 (38%)
Traditional belief	9 (81%)	49 (57%)
Compatibility	2 (19%)	0 (0%)
Accessibility	0 (0%)	2 (2%)
Factors encouraging continued use of *Cajanus cajan* and *Azadirachta indica*:		
Poverty	9 (21%)	8 (9%)
Cultural practice	16 (38%)	47 (46%)
Accessibility	2 (5%)	40 (39%)
Perceived safety	15 (35%)	5 (5%)
Proximity of the plants:		
Near	3 (7%)	83 (81%)
Far	39 (93%)	19 (19%)
Do you know any side effects of *Cajanus cajan* or *Azadirachta indica*?		
Yes	42 (100%)	97 (95%)
No	0 (0%)	5 (5%)
What do you think can encourage people to use prescribed medication more?		
Awareness	26 (62%)	19 (19%)
Subsidy	7 (17%)	68 (67%)
More health centers	0 (0%)	3 (3%)
Public sensitization	9 (21%)	12 (11%)

In the generalized linear model, urban residence was associated with higher odds of herbal use (OR = 3.46, estimate = 1.24, *p* = 0.01). This indicates a statistical association between urban residence and reported herbal use. Occupation was also associated with model fit (*p* = 0.04), with civil servants and farmers showing higher reported use. Age group was associated with herbal use (*p* = 0.01), with the 36–53 year group showing lower odds compared to other age groups (estimate = -1.71, z = -2.78). Overall, variations in residence, occupation, and age were statistically associated with differences in reported herbal use (Table 7).

**Table 7:** Demographic and socio-cultural determinants of knowledge and utilization of *C. cajan* and *A. indica*. The OR = Odds Ratio (e ^ Estimate); CI = Confidence Interval; η²p = Partial Eta Squared (Effect Size). Bold formatting indicates statistically significant predictors (*P* < 0.05 after Bonferroni correction)

Variables	OR	95% CI (OR)	Estimate	SE	z	*P*	η²*p*	95% CI (η²*p*)
Intercept	—	—	—	—	—	—	—	—
Gender	1.15	[0.84, 1.58]	0.14	0.16	0.88	0.16	0.03	[0.00, 1.00]
Resident (Urban)	**3.46**	**[1.30**,** 9.21]**	**1.24**	**0.50**	**2.50**	**0.01**	**0.09**	**[0.02**,** 1.00]**
Occupation						**0.04**	**0.08**	**[0.00**,** 1.00]**
(Civil Servant)	**4.57**	**[1.30**,** 16.03]**	**1.52**	**0.64**	**2.38**	**—**	**—**	**—**
(Farmer)	**5.05**	**[1.31**,** 19.54]**	**1.62**	**0.69**	**2.35**	**—**	**—**	**—**
Age (36–53 yrs)	**0.18**	**[0.05**,** 0.61]**	**-1.71**	**0.62**	**-2.78**	**0.01**	**0.10**	**[0.01**,** 1.00]**
Education	1.28	[0.95, 1.73]	0.25	0.15	1.67	0.10	0.06	[0.00, 1.00]
Age * Education	1.10	[0.98, 1.23]	0.10	0.06	1.67	0.08	0.14	[0.00, 1.00]

Perceptions of efficacy, age, gender, residence and education were associated with treatment choice. Lower perceived efficacy was associated with higher odds of herbal remedy use (OR = 2.41, *p* = 0.012), while moderate perceived efficacy was also associated with herbal use (OR = 2.23, *p* = 0.015). Urban residence was associated with higher odds of herbal remedy use compared with rural residence (OR = 1.63, *p* = 0.006) (Table 8). Tertiary education was also associated with higher odds of herbal remedy use (OR = 2.51, *p* = 0.029). Continued reliance analysis showed that urban residence was associated with higher odds of herbal reliance compared with rural residence (OR = 3.82, *p* = 0.01). Accessibility (OR = 3.16) and high prescription cost (OR = 3.97) were also associated with increased odds of continued herbal use (Table 9).

**Table 8:** Relationship between treatment practices and the perceived efficacy of herbs used for managing malaria, typhoid, and their co-infections. Only statistically significant predictors (*P* < 0.05) are reported. The reference category for treatment practice is prescription. Estimates represent the log-odds of choosing the outcome relative to the reference; OR represents the Odds Ratio

Variables	Outcome Category	Estimate	SE	OR	95% CI	z	*P*
Efficacy (Low)	Herb vs. Prescription	0.88	0.35	2.41	[1.21, 4.80]	2.51	0.012
Efficacy (Moderate)	Herb vs. Prescription	0.80	0.33	2.23	[1.16, 4.25]	2.43	0.015
Age 36–53	Herb vs. Prescription	0.92	0.35	2.51	[1.26, 4.98]	2.61	0.009
Age 54–71	Herb vs. Prescription	0.48	0.17	1.62	[1.16, 2.26]	2.81	0.005
Gender (Male)	Herb vs. Prescription	0.91	0.37	2.48	[1.20, 5.12]	2.48	0.013
Residence (Urban)	Herb vs. Prescription	0.49	0.18	1.63	[1.14, 2.32]	2.75	0.006
Education (Tertiary)	Herb vs. Prescription	0.92	0.42	2.51	[1.10, 5.72]	2.18	0.029
Efficacy (Low)	Prescription vs. Ref.	0.22	0.11	1.25	[1.00, 1.55]	2	0.046
Efficacy (Moderate)	Prescription vs. Ref.	0.90	0.37	2.46	[1.19, 5.08]	2.46	0.014
Age 54–71	Prescription vs. Ref.	0.16	0.07	1.17	[1.02, 1.35]	2.21	0.027
Gender (Male)	Prescription vs. Ref.	0.23	0.10	1.26	[1.03, 1.53]	2.16	0.031
Residence (Urban)	Prescription vs. Ref.	0.79	0.29	2.20	[1.25, 3.89]	2.7	0.007
Education (Secondary)	Prescription vs. Ref.	0.81	0.37	2.25	[1.09, 4.64]	2.18	0.029
Education (Tertiary)	Prescription vs. Ref.	0.62	0.30	1.86	[1.03, 3.35]	2.1	0.036

**Table 9:** Socio-economic and accessibility factors influencing the continued reliance on herbal remedies despite availability of prescription medicines. The OR = Odds Ratio (e^Estimate); CI = Confidence Interval; η²p = Partial Eta Squared (Effect Size). Bold formatting indicates statistically significant predictors (*P* < 0.05 after Bonferroni correction)

Variables	OR	95% CI (OR)	Estimate	SE	z	*P*	η²*p*	95% CI (η²*p*)
Intercept	—	—	—	—	—	—	—	—
Resident (Urban)	**3.82**	**[1.49**,** 9.79]**	**1.34**	**0.48**	**2.76**	**0.01**	**0.08**	**[0.02**,** 1.00]**
Accessibility (Yes)	**3.16**	**[1.28**,** 7.78]**	**1.15**	**0.46**	**2.48**	**0.01**	**0.06**	**[0.01**,** 1.00]**
Cost (Yes)	**3.97**	**[1.58**,** 9.99]**	**1.38**	**0.47**	**2.95**	**0.002**	**0.09**	**[0.02**,** 1.00]**

## Discussion

In this study examining factors influencing the indigenous use of *C. cajan* and *A. indica* for managing typhoid, malaria, and their co-occurrence in rural and urban areas of Nsukka, Nigeria, we found that age distributions were broadly similar across both settings, with respondents aged 36–53 forming the largest group of participants. There were also clear socio-economic differences: most urban respondents were civil servants or business workers and had predominantly attained tertiary education, whereas rural respondents were mainly farmers and herbalists with mainly primary-level education (Table 1).

**Fig. 2:**
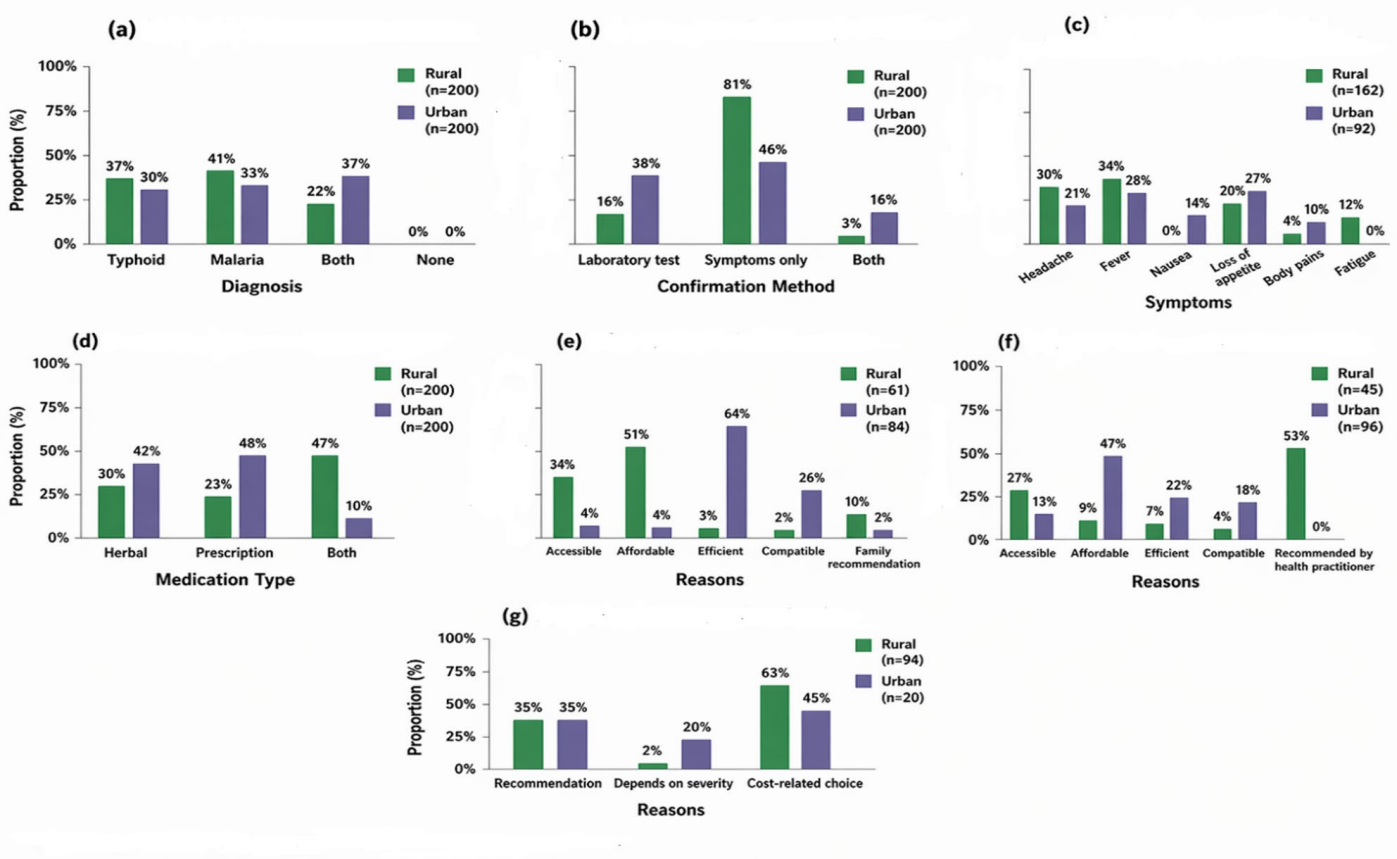
Respondents’ knowledge, practices and treatment preferences regarding typhoid and malaria in urban and rural Nsuka. **A** Diagnosis history; **B** Methods of confirming infection; **C** Symptoms experienced among respondents relying on symptom-based diagnosis; **D** Type of medication used for prevention or treatment; **E** Reasons for preferring herbal medicine; **F** Reasons for preferring prescription medicine; **G** Reasons for combined use of herbal and prescription treatments (*n* = Urban, 200; Rural, 200)

Based on our hypotheses, we expected that socio-demographic factors (education and residence), as well as accessibility-related factors (cost of medication, healthcare access, and knowledge of safe herb–drug use), would significantly influence knowledge and use of herbal medicine and reliance on herbal remedies in rural and urban settings. However, our findings provided only partial support for these hypotheses, as education level did not emerge as a significant predictor. Instead, the results indicate that occupation, age, and residence are the predominant demographic and socio-cultural drivers associated with the knowledge and utilization of *C. cajan* and *A. indica* (Table 1). Interestingly, urban dwellers were more likely than rural residents to report using herbs and prescription drugs separately or in combination during management of malaria, typhoid and co-infections (Table 7; Fig. 2). These findings may reflect greater access to healthcare and pharmaceutical outlets in urban areas, enabling residents to obtain prescription medicines alongside traditional remedies. Although increased herbal use has been linked to self-medication practices [34], it is also plausible that some urban dwellers prefer herbal remedies due to reluctance to disclose private health concerns to physicians or pharmacists because of perceived confidentiality concerns [35].

More so, while descriptive data suggest that rural respondents maintain a stronger historical and family-based reliance on herbal extracts (Tables 3 and 4), the GLM indicates that urban residence was significantly associated with higher reported utilization of herbal medicine (Table 7). This apparent discrepancy reflects the distinction between descriptive distributions of knowledge and use and model-adjusted associations controlling for socio-demographic variables. Rural participants tended to use herbal extracts mainly for acute treatment, whereas urban respondents more frequently reported prophylactic use (90%) and use as an alternative when perceived prescription efficacy was low (Tables 3 and 5). Thus, urban residence does not necessarily reflect limited access to biomedical care but is associated with distinct patterns of preventive and complementary herbal use within a pluralistic healthcare context (Table 7). The discrepancy between descriptive patterns and GLM outputs therefore reflects differences between raw prevalence estimates and adjusted multivariable associations.

**Fig. 3:**
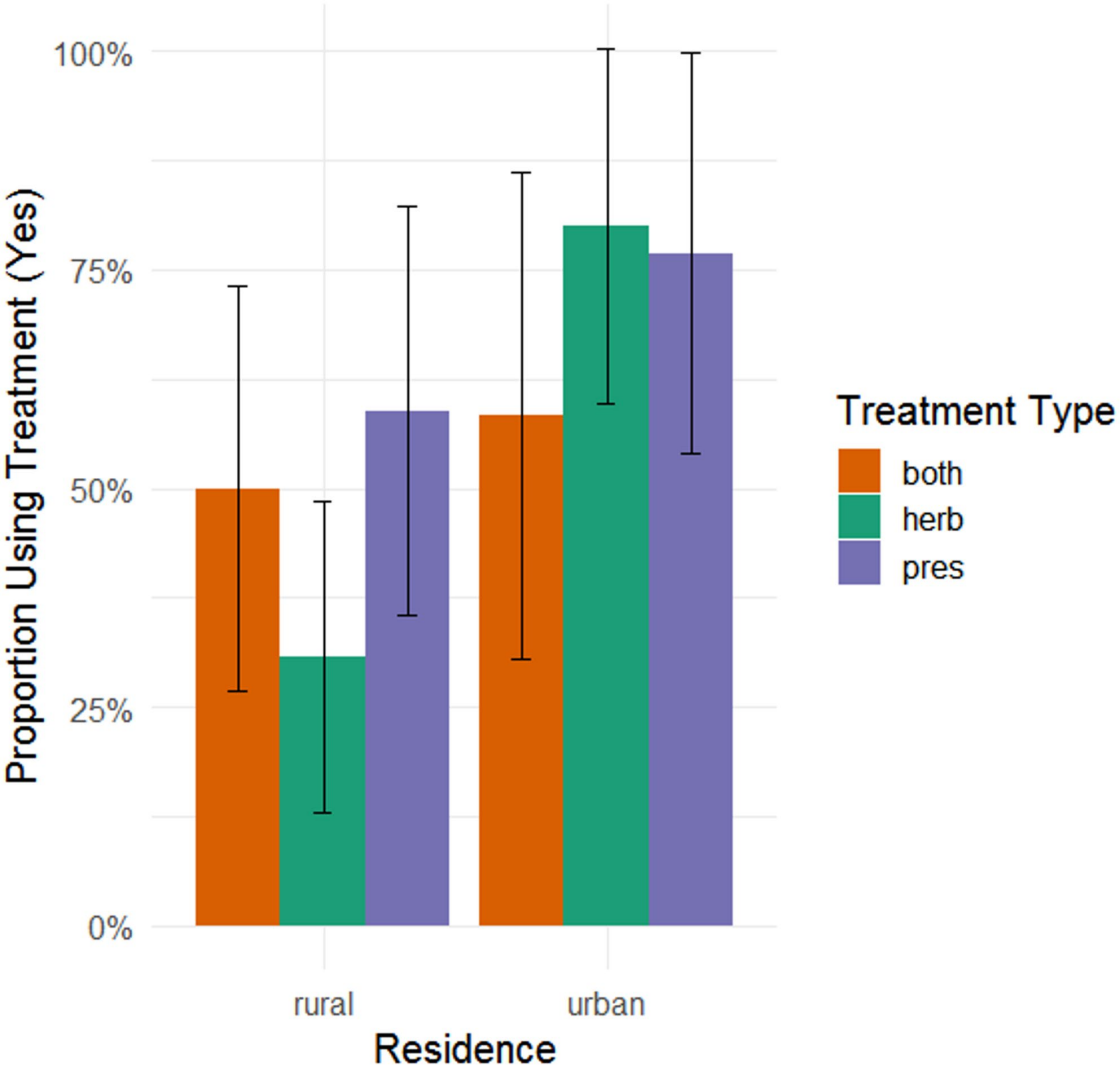
Use of herbal, prescription, and combined remedies by residents

In this study, education alone was not significantly associated with knowledge and use; however, the interaction between education level and age range appeared to be a stronger contributor (partial N² = 0.14; Table 7). This suggests that different age groups interpret and apply herbal knowledge differently depending on educational exposure and that herbal use is shaped by combined socio-cultural influences rather than single demographic variables. In line with this, higher formal education among urban respondents (many of whom attained tertiary education: Table 1) may be associated with greater awareness of available treatment options and the adoption of multiple therapies to maximize perceived benefit [36], as reflected in Fig. 3. Meanwhile, occupation was associated with herbal knowledge and use, suggesting that civil servants and farmers differ in access to resources, familiarity with medicinal plants, or exposure to healthcare systems (Table 7).

**Fig. 4:**
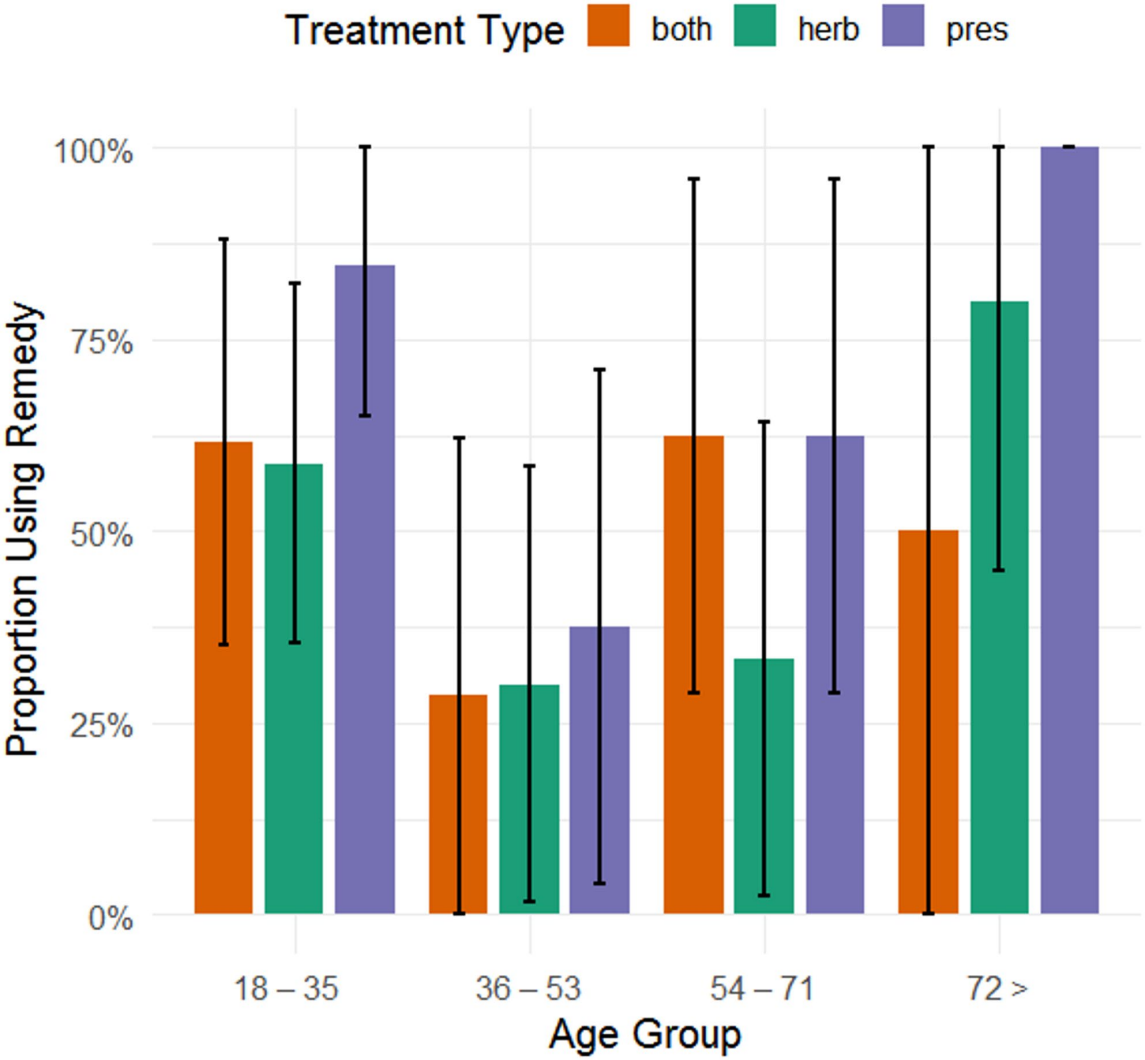
Age-wise use of herbal, prescription, and combined remedies by respondents

We found that both urban and rural respondents reported high exposure to malaria and typhoid, with malaria slightly more common in rural areas, while urban respondents reported more co-occurrence (Fig. 4a). The high prevalence of malaria and its co-occurrence with typhoid aligns with previous findings [37]. Our study also indicated that laboratory confirmation was more common in urban areas, whereas rural respondents largely relied on symptom-based diagnosis (Table 2; Fig. 4b), likely reflecting limited access to diagnostic facilities in rural communities. Among those relying on symptoms, fever and headache were the most frequently cited indicators across both settings (Fig. 4c). Nonetheless, limited public health infrastructure, including diagnostic services, may increase vulnerability to self-diagnosis and self-treatment practices [38]. We also observed that urban respondents more frequently reported prescription medicine use, while rural respondents more often reported reliance on herbal remedies or combined approaches (Fig. 4d), likely influenced by affordability, tradition, and accessibility (Table 2). Proximity to sources of *A. indica* and *C. cajan* was higher in rural areas (Table 6), which may be associated with these observed usage patterns.

Despite the availability of prescription medicines, accessibility and cost were strongly associated with continued herbal use (Table 9). In both urban and rural settings, respondents with easier access to herbs were more likely to report using them, while cost remained a major factor associated with treatment choice (Tables 2 and 9). These findings indicate that socio-economic and spatial factors jointly influence treatment practices, where availability and affordability reinforce reliance on traditional medicines (Fig. 4e-g). Previous studies similarly report that herbal medicine use increases with proximity and accessibility of plant sources [39]. Nonetheless, these results provide strong support for our fourth (accessibility factors), as proximity and cost consistently influenced reliance on herbal medicine. We expected that long-standing family traditions would be associated with dosage practices, perceived efficacy, and continued use of medicinal plants, and that treatment preference would be associated with perceived efficacy and frequency of use. Consistent with this, rural respondents reported acquiring knowledge of *A. indica* and *C. cajan* primarily through family history (Tables 3 and 4). Family recommendations were also associated with use during co-occurrence of malaria and typhoid (Table 5). Herbs were mainly prepared as decoctions, with respondents reporting perceived relief and few side effects. This reflects intergenerational transmission of knowledge, particularly in rural communities where older family members often serve as trusted sources of treatment guidance. Rural respondents also showed more consistent adherence to traditional preparation methods and longer treatment durations, which may reflect stronger cultural influence and limited exposure to alternative biomedical guidance. These patterns suggest that family-based knowledge is associated with preparation and dosage practices and sustained perceived effectiveness (Tables 3, 4, 5 and 6).

Additionally, treatment preference was associated with perceived efficacy (Table 8). Respondents using herbal remedies were more likely to report low or moderate perceived efficacy compared with those using prescription drugs. This outcome supports our third (treatment modality and perceived effectiveness), although the direction of association suggests variability in perceived outcomes depending on preparation methods, dosage differences and illness type, however, non-standardized preparation and dosage have previously been identified as key limitations of herbal medicine effectiveness [40, 41]. In contrast, urban residence, herb accessibility and cost were associated with higher likelihood of herbal use (Table 9), where respondents reported that they use herbal remedies because they are affordable, easily accessible and perceived as compatible with their socio-economic conditions. Probably because herbal remedies are widely perceived as safe alternatives, particularly in contexts where concerns about side effects of synthetic drugs and high prescription costs exist [42, 43].

While most studied factors are associated with continued herbal use even when biomedical options are available, the concurrent use of *C. cajan* and *A. indica* alongside prescription medicines should raise important considerations regarding potential herb–drug interactions. Indeed, participants reported perceived safety and effectiveness in combining both herbs and prescribed drugs, but then it is essential to note that most of these plants also contain bioactive compounds such as flavonoids, alkaloids and terpenoids that may theoretically interact with conventional antimalarial and antibiotic agents via pharmacokinetic or pharmacodynamic pathways that may potentially affect drug absorption, metabolism or therapeutic efficacy. In turn, such may result in either reduced drug effectiveness or, in some cases, enhanced pharmacological effects depending on dosage, formulation and timing of administration. However, since this study did not include laboratory-based toxicological or pharmacological assessments; there is no definitive conclusions drawn regarding safety or interaction outcomes. But nonetheless, our findings still highlight the need for further experimental and clinical studies to evaluate the safety profile and potential interaction risks associated with the combined use of herbal remedies and prescription drugs within this population.

## Conclusion

This study shows that the use of *C. cajan* and *A. indica* for managing malaria, typhoid, and their co-occurrence in Nsukka is mainly associated with residence, occupation, age, accessibility, and cost rather than education alone. Rural respondents reported greater reliance on herbal remedies, largely linked to affordability, proximity to plant sources, and strong family traditions, while urban respondents more frequently reported using herbs alongside prescription medicines due to greater access to healthcare services and pharmaceuticals. Family-based knowledge was associated with preparation methods, dosage practices, and perceived effectiveness of these medicinal plants, supporting their continued use across both settings. Variations in preparation and dosage practices were associated with differences in perceived effectiveness, while accessibility and cost consistently influenced treatment choices. Nonetheless, herbal medicine use in the study area is shaped by the interaction of socio-cultural knowledge systems, economic constraints, and healthcare access rather than any single demographic factor. These findings highlight the importance of providing context-specific public health guidance and supporting the safe and coordinated use of traditional and conventional treatment practices within primary healthcare systems.

### Strengths and limitations

In spite the outcome of this study, it is important to note that interpretations regarding illness status and treatment effectiveness are based on self-reported data rather than laboratory-confirmed diagnoses or standardized clinical measurements. Therefore, findings related to malaria and typhoid occurrence, as well as perceived efficacy of herbal remedies, should be interpreted as reported experiences rather than clinically verified outcomes. In addition, variability in herbal preparation and dosage was not experimentally controlled and may have influenced reported treatment responses. For these reasons, the present study has several limitations that should be considered when interpreting the findings. First, all disease status, herbal use and treatment practices were based on self-reported information, which may be subject to recall bias, particularly regarding past illness episodes, duration of use and frequency of herbal consumption. Also, social desirability bias may have influenced responses, as participants might have over reported culturally accepted herbal practices or underreported reliance on prescription medicines. Second, the study employed a cross-sectional design, which limits the ability to establish temporal relationships between variables and precludes causal inference; findings therefore reflect associations rather than cause-and-effect relationships. More so, there was no laboratory confirmation of malaria or typhoid infections, as diagnoses were based on participant-reported symptoms or prior clinical suspicion, which may have introduced misclassification bias. Current study also did not include biological or pharmacological validation of the efficacy of *C. cajan* and *A. indica*, and therefore all interpretations regarding effectiveness are based on perceived outcomes rather than clinical verification. In addition, there was no standardization or quantitative measurement of herbal dosage, preparation methods, or concentration, which may contribute to variability in reported usage patterns. Despite these limitations, the study provides important insight into socio-cultural and socio-economic determinants of herbal medicine use in Nsukka, Nigeria, and contributes to understanding patterns of interaction between traditional and biomedical treatment systems.

### Recommendations

Based on the findings of this study, several recommendations are proposed to improve the safe and effective use of *Cajanus cajan* and *Azadirachta indica* in the management of malaria, typhoid, and their co-occurrence. First, there is a need for structured dialogue between traditional medicine practitioners and primary healthcare providers to promote safer and more coordinated patient care. Such engagement would help bridge communication gaps, improve referral pathways, and support informed decision-making regarding the concurrent use of herbal and prescription medicines. Second, public health authorities should implement community-based health education programmes focusing on the safe use of medicinal plants, appropriate timing and combination with conventional drugs, and awareness of potential herb–drug interaction risks, particularly given the high prevalence of combined use observed across settings. Third, there is a need for capacity-building and training of healthcare workers on commonly used herbal medicines within the study area, enabling them to better advice patients who use both systems of care. Fourth, regulatory and health agencies should encourage the development of collaborative frameworks for documenting and standardizing traditional herbal practices, including preparation methods and dosage guidelines, to reduce variability and improve safety. Finally, further pharmacological and clinical studies are recommended to evaluate the efficacy, safety, and potential herb–drug interactions of *C. cajan* and *A. indica*, particularly in relation to commonly used antimalarial and antibiotic therapies.

## Supplementary Information

Supplementary Material 1.

## Data Availability

All data will be available from the corresponding author upon reasonable request.

## Abbreviations

GLMs: Generalized linear models
MLR: Multinomial logistic regression
*C cajan*: Cayanus cajan
*A. indica*: Azaridacta indica
