# Supplementary material for: Family traditions, accessibility and cost shape the continued reliance on Cajanus cajan and Azadirachta indica for malaria and typhoid despite prescription medicines in urban and rural Nsukka, Nigeria
Source: BMC Complement Med Ther. 2026 Jun 4;26:239. doi: 10.1186/s12906-026-05426-y (PMC13449695; doi:10.1186/s12906-026-05426-y)
Supplement: Supplementary file 1 [file 12906_2026_5426_MOESM1_ESM.docx]

**Indigenous Use of pigeon pea (*Cajanus cajan*) and neem (*Azadirachta indica*) for the Management of Typhoid, Malaria, and Their Co-occurrence in Nsukka, Nigeria****

Introduction

This survey is designed to capture current knowledge and usage of *Cajanus cajan* (pigeon pea) and *Azadirachta indica* (neem) as alternate and traditional medications for typhoid and malaria management in rural and urban areas of Nsukka LGA. It targets individuals aged 18 years and above. Respondents are required to choose only one answer per question, unless otherwise stated. All information provided will be treated as confidential and used strictly for research purposes.

**Section A: Respondent Demographics**

1. Gender:

a. Male

b. Female

2. Age range:

a. 18–35

b. 36–53

c. 54–71

d. 72 and above

3. Marital status:

a. Yes

b. No

4. If married, number of children:

a. None

b. 1–5

c. 6–10

5. Occupation:

a. Civil servant

b. Public servant

c. Business

d. Farmer

e. Herbalist

6. Level of education:

a. None

b. Primary

c. Secondary

d. Tertiary

7. Place of residence:

a. Urban

b. Rural

**Section B: Knowledge about Typhoid and Malaria**

8. Have you ever been diagnosed or believed to have:

a. Typhoid

b. Malaria

c. Both

d. None

9. How do you confirm typhoid or malaria infection?

a. Laboratory test

b. Symptoms only

c. Both

10. If based on symptoms, which do you usually experience?

a. Headache

b. Fever

c. Nausea

d. Loss of appetite

e. Body pains

f. Fatigue

11. Type of medication used for prevention or treatment:

a. Herbal

b. Prescription

c. Both

12. If herbal, why do you prefer it?

a. Accessible

b. Affordable

c. Efficient

d. Compatible

e. Family recommendation

13. If prescription, why do you prefer it?

a. Accessible

b. Affordable

c. Efficient

d. Compatible

e. Recommended by health practitioner

14. If both, which do you use more often?

a. Herbal

b. Prescription

c. Depends on severity

d. Cost-related choice

**Section C: Knowledge and Use of *Cajanus cajan***

15. Do you have any knowledge about *Cajanus cajan*?

a. Yes

b. No

16. If yes, what is the plant mostly used for?

a. Malaria

b. Typhoid

c. Ulcer

d. Purgative

17. If used for typhoid, how did you acquire this knowledge?

a. Family

b. Recommendation

c. Books

d. School

e. Herbal practitioner

18. Frequency of *Cajanus cajan* use for typhoid:

a. Always

b. Rarely

c. Never

19. Duration of use (years):

a. 1–5

b. 6–10

c. 11–20

d. Above 20

20. When is *Cajanus* *cajan* mainly used?

a. Prevention

b. Treatment

c. Both

21. Have you alternated *Cajanus cajan* with prescribed medications?

a. Yes

b. No

22. If yes, how do you alternate them?

a. *Cajanus cajan* for prevention, prescribed for treatment

b. *Cajanus cajan* for treatment, prescribed for prevention

c. Use both together

23. How is *Cajanus cajan* usually prepared?

a. Boiling leaves or roots (decoction)

b. Soaking (infusion)

c. Grinding with water

d. Mixing with other herbs

e. Other (specify) __________

24. Usual dosage or rate of consumption:

a. One cup (≈250 ml) twice daily

b. One cup once daily

c. Half cup twice daily

d. Other (specify) __________

25. Duration per treatment cycle:

a. 1–3 days

b. 4–7 days

c. More than 7 days

26. Experienced side effects after using *Cajanus cajan*:

a. Yes

b. No

If yes, specify: __________________________

**Section D: Knowledge and Use of *Azadirachta indica***

27. Do you have any knowledge about *Azadirachta indica*?

a. Yes

b. No

28. If yes, what is it mostly used for?

a. Malaria

b. Typhoid

c. Both

d. Other (specify) __________

29. Source of knowledge

a. Family

b. Traditional healers

c. Books

d. School

e. Observation

30. Frequency of neem use for malaria:

a. Always

b. Occasionally

c. Never

31. Part of neem used:

a. Leaves

b. Bark

c. Roots

d. Seeds

e. Combination

32. Preparation method:

a. Boiling (decoction)

b. Soaking (infusion)

c. Mixing with other herbs

d. Grinding

e. Other (specify) __________

33. Administration method:

a. Drink

b. Bath

c. Steam inhalation

d. Other (specify) __________

34. Dosage/consumption rate:

a. One cup (≈250 ml) twice daily

b. One cup once daily

c. Half cup twice daily

d. Other (specify) __________

35. Duration of use per treatment:

a. 1–3 days

b. 4–7 days

c. More than 7 days

36. Perceived efficacy of neem:

a. Very effective

b. Effective

c. Slightly effective

d. Not effective

37. Experienced side effects:

a. Yes

b. No

If yes, specify: __________________________

**Section E: Co-Occurrence (Typhoid and Malaria Together)**

38. Have you ever experienced typhoid and malaria at the same time?

a. Yes

b. No

39. If yes, how do you usually treat both?

a. *Cajanus cajan* and *Azadirachta indica* together

b. *Cajanus cajan* only

c. *Azadirachta indica* only

d. Combine herbs with hospital drugs

40. Preparation method when combining herbs:

a. Separately

b. Together in same decoction

c. Sequentially (one after another)

41. Dosage when combining herbs:

a. One cup (≈250 ml) twice daily

b. One cup once daily

c. Half cup twice daily

d. Other (specify) __________

42. Relief experienced when using both:

a. Yes

b. No

c. Partially

43. Who recommends the use of these herbs for co-occurrence?

a. Family

b. Herbalist

c. Friends

d. Self

e. Others

44. Experienced side effects when using both herbs together:

a. Yes

b. No

If yes, specify: __________________________

**Section F: Factors Influencing Herbal Use**

45. Factors influencing preference for herbal remedies: (Select all that apply)

a. Cost

b. Accessibility

c. Cultural belief

d. Effectiveness

e. Family influence

46. Would you stop using herbal medicines if prescribed drugs were more affordable?

a. Yes

b. No

47. If no, why do you still prefer herbal remedies?

a. Affordability

b. Cost-effective

c. Traditional belief

d. Compatibility

e. Accessibility

48. Factors encouraging continued use of *Cajanus cajan* and *Azadirachta indica*:

a. Poverty

b. Cultural practice

c. Accessibility

d. Perceived safety

49. Proximity

a. Near

b. Far

**Section G: Awareness and Safety**

49. Do you know any side effects of *Cajanus cajan* or *Azadirachta indica*?

a. Yes

b. No

50. If yes, which ones?

a. Constipation

b. Nausea

c. Infertility

d. Dizziness

e. Kidney stones

f. None

g. Other (specify) __________

51. Ever been advised by a medical practitioner against using these herbs?

a. Yes

b. No

52. What do you think can encourage people to use prescribed medication more?

a. Awareness

b. Subsidy

c. More health centres

d. Public sensitization

53. Best way to prevent malaria and typhoid:

a. Good sanitation

b. Use of medication

c. Use of insecticide-treated nets

d. Both sanitation and treatment
